# *Lactobacillus crispatus* thrives in pregnancy hormonal milieu in a Nigerian patient cohort

**DOI:** 10.1038/s41598-021-96339-y

**Published:** 2021-09-13

**Authors:** Nkechi Martina Odogwu, Chinedum Amara Onebunne, Jun Chen, Funmilola A. Ayeni, Marina R. S. Walther-Antonio, Oladapo O. Olayemi, Nicholas Chia, Akinyinka O. Omigbodun

**Affiliations:** 1 Pan African University Life and Earth Sciences Institute (PAULESI), University of Ibadan, Ibadan, Oyo State Nigeria; 2 Division of Surgical Research, Department of Surgery, Mayo Clinic, Rochester, MN USA; 3 Microbiome Program, Center for Individualized Medicine, Mayo Clinic, Rochester, MN USA; 4 Department of Obstetrics and Gynecology, University College Hospital, Ibadan, Nigeria; 5 Department of Health Science Research, Mayo Clinic, Rochester, MN USA; 6 Department of Pharmaceutical Microbiology, Faculty of Pharmacy, University of Ibadan, Ibadan, Nigeria; 7 Department of Obstetrics and Gynecology, Mayo Clinic, Rochester, MN USA; 8 Department of Obstetrics and Gynecology, College of Medicine, University of Ibadan, Ibadan, Nigeria; 9 Division of Clinical Microbiology, Department of Laboratory Medicine and Pathology, Mayo Clinic, Rochester, MN USA

**Keywords:** Microbiology, Molecular biology, Endocrinology, Medical research

## Abstract

Steroid hormones are one of the presumed modulators of *Lactobacillus* abundance in the vaginal epithelium. We set out to characterize the vaginal microbiome (VMB) and also provide an in-depth understanding of the relative contribution of estradiol (E2) and progesterone (P1) in shaping the vaginal microbiome of Nigerian women (n = 38) who experienced both uncomplicated term delivery and preterm delivery using samples longitudinally collected during pregnancy (17–21, 27–31, 36–41 weeks gestation) and 6 weeks postpartum. Vaginal swabs and blood samples were aseptically collected. Vaginal swabs were used for microbiome assessment using 16S ribosomal RNA (rRNA) gene sequencing. Blood samples were used for hormonal measurement using a competitive-based enzyme-linked immunosorbent assay (ELISA). Across several maternal covariates, maternal age, pregnancy status and delivery mode were not significantly associated with the vaginal microbiota whereas maternal E2 level (p_E2_ = 0.006, Omnibus), and P1 level (p_P1_ = 0.001, Omnibus) were significantly associated with the vaginal microbiome. E2 and P1 concentrations increased throughout pregnancy commensurately with increasing proportions of *L. crispatus* (p_E2_ = 0.036, p_P1_ = 0.034, Linear Mixed Model). An increasing trend of α-diversity was also observed as pregnancy progressed (p_observed ASV_ = 0.006, LMM). A compositional microbiome shift from *Lactobacillus* profile to non-*Lactobacillus* profile was observed in most postnatal women (p_CST IV_ < 0.001, LMM). Analysis of our data shows a species-specific link between pregnancy steroid hormone concentration and *L. crispatus* abundance.

## Introduction

The vaginal microbiome in pregnancy has been found to be involved in both maternal health outcome^1–3^ and disease pathogenesis^4–7^. Vaginal microbial communities state types (CSTs) are typically defined by the dominance of one of four *Lactobacillus* species; *L*. *crispatus* (CST I), *L. gasseri* (CST II), *L. iners* (CST III) and *L. jensenni* (CST V) while CST IV is defined by heterogenous mixture of strict and facultative anaerobes^8^. In pregnancy, healthy obstetric outcomes are associated with an enrichment of *Lactobacillus* and low bacterial diversity^1,3,9^. In this context, *Lactobacillus* are associated with full-term birth and modulate reproductive health^10,11^ through the production of lactic acid^12,13^ and antimicrobial compounds^14^. The presence of different Lactobacillus species in the vaginal microenvironment is widely believed to enhance stability of the vaginal microbiome in pregnancy as failure of these species to retain dominance over time (remain stable) may result to overgrowth of anaerobic bacteria including bacterial vaginosis associated bacteria (BVAB1), *Prevotella species, Gardnerella vaginalis, Atopobium vaginae, Sneathia, Megasphaera*^15^. These anaerobes have been shown to be associated with poor pregnancy outcomes such as post-abortal infections, early miscarriage and preterm birth^16–18^. It is important to consider the microbiome in health disparities as recent studies also reveal that women of African ancestry are less likely to exhibit vaginal lactobacilli, frequently have less stable VMB, are more likely to exhibit increased vaginal microbial diversity and richness^19–22^.

The composition of the vaginal microbiome appears hormonally regulated^23^ as evidence shows it adapts to hormonal changes in menstruation^24^ and menopause^25^. In general, high steroid hormone levels tend to decrease the dominance of anaerobic bacteria and favor the dominance of *Lactobacillus* on the vaginal epithelium^26,27^. Pregnancy is accompanied by a rise in the level of estrogens and progesterone hormones^28^, thus as pregnancy advances, elevated levels of circulating estrogen are hypothesized to increase Lactobacilli species abundance. The withdrawal of estrogen during the postpartum period is also assumed to precipitate a dramatic shift from a prior *Lactobaccillus* dominated microbiota to a diverse and non-*Lactobacillus* dominated microbial profile in postnatal women^2,29^. Several studies of North American^1,6,9,29,30^ and European populations^2^ have characterized the vaginal microbiota throughout pregnancy. These studies show the vaginal microbiomes of pregnant women to be highly stable and typically dominated by *Lactobacillus*. Unfortunately, these studies can provide us with little insight about the dynamics of the pregnancy vaginal microbiome with the shifting hormonal milieu during the postpartum period. In addition, ethnicity is also an important determinant of the vaginal microbiome composition during pregnancy as Black ethnicity is a risk factor for bacterial vaginosis^31^. In Africa, the vaginal microbiome during pregnancy has been described in an HIV pregnant cohort^32,33^ but only using a cross sectional study design. To our knowledge, this is the first study describing the vaginal microbiota during pregnancy and postpartum longitudinally. Using this data, we set out to characterize the VMB during pregnancy and puerperium in Nigerian women and provide an in-depth understanding of the relative contribution of estradiol (E2) and progesterone (P1) in shaping the vaginal microbiome.

## Methods

### Ethical statement and participant enrollment

Ethical approval for this study was obtained in accordance with the Nigerian National Code for Health Research Ethics and approved by the Joint University of Ibadan/University College Hospital Health Research Ethics Committee (UI/UCH HREC) with approved IRB number UI/EC/18/0411. All experiments were performed in accordance with the approved guidelines. Written informed consent was obtained from all participants prior to sampling. Women (n = 38) aged between 24 and 41 years who had no medical problems or adverse outcome during any previous pregnancy were enrolled to the longitudinal study at the time of booking for antenatal care. Participants were recruited from 17 weeks’ gestation, getting details of their previous pregnancies history and other maternal and fetal covariates and monitoring them until puerperium (6 weeks postpartum). Biological samples were collected thrice during pregnancy: at the recruitment visit occurring between 17 and 21 weeks (first timepoint), at 27–31 weeks (second timepoint), above 36–41 weeks’ gestation (third timepoint), and once at 6 weeks postpartum (fourth time point). Women were included in the study if they: self-reported as Nigerian; they were between 17 and 21 weeks’ gestation (confirmed by clinical records and ultrasound result); were of reproductive age (18–49 years); have no intercurrent infection requiring treatment with antibiotics, weighed greater than 50 kg; had no medical complication during any previous and current pregnancy including diabetes mellitus, autoimmune disease and hypertension; were not on supplemental progesterone, were able to provide written informed consent; and were willing to participate in all aspects of the study. Women were excluded if they: developed any inter-current infection requiring antibiotic therapy; had sexual activity within 72 h of sampling; used probiotic supplement, medication or probiotic feed in the preceding 2 weeks before sampling, reported vaginal bleeding in the preceding week, used antibiotics in the preceding 2 weeks, had chronic active viral infections, including HIV-1/2, HTLV-1/2, hepatitis B/C or have known autoimmune disease, such as, rheumatoid arthritis or systemic lupus erythematosus; solid organ or transplant recipient as previously described^34^. To reduce incidence of vaginal infection induced by douching agents or preterm birth induced by douching agent we excluded women who use vaginal douching. These foundational data were collected by the research team and participants were followed up at every antenatal visit taking details of important maternal and clinical variables until delivery (both term and preterm).

### Sample collection

Blood and vaginal swabs were simultaneously collected during sampling. Under visual inspection during a speculum examination, swabs (LQ AMIES Copan CA, USA) were collected from the posterior fornix^35^. The vaginal swabs for genomic bacterial DNA isolation were transferred to the laboratory in Amies transport medium and stored at − 80 °C until isolation. Three milliliter of blood samples were collected aseptically from the peripheral vein of the arm in an EDTA vacutainer blood collection tube and transported on ice to the laboratory for further analysis.

### Sample processing

Collected blood samples were transported on ice and processed within one hour of collection. Blood samples were centrifuged at 3000 rpm for 10 min (Unico Powerspin Hxdb Centrifuge, NY, USA). The supernatant (plasma) was decanted and stored at -80 °C until assay.

### Estradiol and progesterone analysis

Plasma samples from each participant were thawed at room temperature and assayed together on the same day in one batch to eliminate between-assay variability. Plasma concentrations of both hormones were quantified by a high-performance solid phase competitive enzyme-linked immunosorbent assay (Human estradiol and Progesterone ELISA Kit, Calbiotech, Inc, Cordell CA, USA) on an ELISA Reader (Thermofischer Scientific) as previously described^36^.

### DNA extraction

Genomic DNA was extracted from stored vaginal swabs using the Zymobiomics DNA extraction Kit (Zymo research Irvine, CA USA) adhering to the manufacturer’s protocol as previously described^37^. Briefly, frozen swabs were thawed on ice and suspended into a 2 ml bashing bead lysis tube containing lysis solution. Microbial cells were lysed by mechanical disruption with a high-speed cell disruptor (FastPrep Classic Instrument, MP Biomedicals, LLC Irvine, CA, USA) set at 6.0 m/s for 1 min. The Bashing Bead lysis tube were centrifuge at 10,000 × *g* for 1 min. The resulting lysate (supernatant) was further processed with the Zymobiomics DNA extraction Kit (Zymo research Irvine, CA USA) and the DNA was eluted in 100 μl of TE buffer.

### Vaginal 16S rRNA gene amplification and sequencing

Amplification of the V3–V5 hypervariable regions of the 16S rRNA gene was performed using a two step-PCR and then incorporating Illumina flow cell adaptors containing indices as previously described^38^. In the first PCR reaction samples were amplified with the following conditions: 95 °C for 5 min, 35 cycles of: 98 °C for 20 s, 55 °C for 19 s, and 72 °C for 60 s, a final 72 °C extension for 5 min and hold at 4 °C.

V3_515F and V5_806R primers^39^ modified with Nextera adaptors were developed in collaboration with the University of Minnesota Genomic Center in Minneapolis, MN, USA.

V3_515F_Nextera:

TCGTCGGCAGCGTCAGATGTGTATAAGAGACAGCCTACGGGAGGCAGCAG

V5_806R_Nextera:

GTCTCGTGGGCTCGGAGATGTGTATAAGAGACAGCCGTCAATTCMTTTRAGT

Primary PCR products were diluted 1:100 in PCR grade water for secondary PCR reactions. PCR cycling conditions were 95 °C for 5 min, 10 cycles of: 98 °C for 20 s, 55 °C for 15 s, and 72 °C for 60 s, and a final 72 °C extension for 5 min. The second amplification was performed using different combinations of forward and reverse indexing primers. The following indexing primer design was utilized^38^, ([i5] and [i7] indicates the position of the forward and reverse indices respectively.

Forward i5 primer: AATGATACGGCGACCACCGAGATCTACAC[i5]

TCGTCGGCAGCGTC

Reverse i7 primer: CAAGCAGAAGACGGCATACGAGAT[i7]

GTCTCGTGGGCTCGG

PCR products were diluted to 20 μL with PCR grade water and cleaned up using 1.0× AMPureAP beads (Beckman Coulter, Brea, CA), vacuum-dried, reconstituted in 12 μL of PCR grade water, quantified using a Quant-It dsDNA HS assay kit (Thermo Fisher Scientific Inc., Waltham, MA), normalized and pooled. The sequencing pool was concentrated, cleaned up using 1.8× AMPureAP beads (Beckman Coulter, Brea, CA). Pooled 16S amplicon samples are quantified using the KAPA SYBR FAST qPCR kit (KAPA Biosystems, Woburn, MA), diluted to 2 nM, denatured with an equal volume of 0.2 N NaOH, diluted to 8 pM with Illumina HT1 buffer, spiked with 10% PhiX, heat denatured at 96 °C for 2 min immediately prior to loading and sequenced using the MiSeq 600 cycle v3 kit (Illumina, San Diego, CA) and MCS v2.6.1.

### Bioinformatic methods for microbiota analysis

The sequence reads were processed using the QIIME 2 bioinformatic pipeline (2019 v7)^40,41^. Raw paired end demultiplexed sequence were quality filtered and adapter trimmed. Further processing followed the DADA2 workflow where sequencing reads were denoised to form amplicon sequence variants (ASVs)^42^. A reference data base, SILVA database v132 was used for taxonomic assignment^43^. Read counts for ASVs assigned to the same taxonomy were summed for each sample. All samples were analyzed in the same run to reduce batch effects that may arise from run-to-run variability**.** A phylogenetic tree was constructed using FastTree v2.1^44^. ASVs with otherwise unidentified genus and species were identified by using BLAST to query their corresponding representative genomes. Representative BLAST matches were chosen if they achieved a query cover and percentage identity of 100% respectively. To normalise our data, a sampling depth of 1208 sequence read per sample was used. Artifact, non-bacterial and singleton ASVs were also removed^45^. Community state types (CST) were assigned to each sample based on ASV abundance using hierarchical clustering with Hellinger distance (Euclidean distance on square-root proportion data) and Ward linkage as described previously^46^. Each CST is defined by the dominance of *L*. crispatus (CST I), *L. gasseri* (CST II), *L. iners* (CST III), an heterogenous mixture of strict and facultative anaerobes (CST IV) comprising of CST IV-A and CST IV-B and *L. jensenni* (CST V) as previously described^8,47^. Tables comprising of distribution of samples after quality control and total sequence data for each sample with more than 1208 sequences were generated and used for statistical analyses. A total of 1,067,231 sequences were generated from 87 samples for an average of 12,039 reads per sample (Figure S1).

### Statistical analysis

Custom scripts written in the statistical language R were used for statistical analysis^48^. Significant differences between participants clinical characteristics were determined using two-tailed Student’s t test, Wilcoxon rank sum test or Fisher’s-exact test where appropriate. Analysis of microbiome data was performed for α-diversity, β-diversity and taxa abundances. For α-diversity and β-diversity analyses we rarefied the sequence data to a common depth of 1208 reads. Briefly, α-diversity reflects species richness and evenness within bacterial populations^49^. Two α-diversity metrics, the observed ASV number and the Shannon index, were calculated on the rarefied dataset. The observed ASV number reflects species richness, whereas the Shannon index reflects species’ richness and evenness. To test the longitudinal association between the covariates and α-diversity, a linear mixed effect model (LMM) was used while adjusting for potential confounders where necessary. β diversity reflects the similarities or shared diversity between bacterial communities in terms of ecological distance between samples^49^. Four β diversity measures (unweighted, generalized (α = 0.5), weighted UniFrac distances and Bray–Curtis distance) were calculated^50^. The unweighted UniFrac reflects differences in community membership (i.e., the presence or absence of an ASV), whereas the weighted UniFrac captures both differences in community membership and also reflects differences in abundance. The Bray–Curtis quantifies the compositional dissimilarity between the two groups based on counts of each sample across each group^51^. The Augmented Dickey-Fuller Test, was used to identify stable taxa across all pregnancy samples^52^, previously described in^53^. The quantitative measure of community stability was estimated with the UniFrac distance measure (both weighted and unweighted). To test the association between the steroid hormones (estradiol and progesterone), other covariates including age groups (age above 35 and age below 35), delivery mode (ceaserian section and vaginal delivery, pregnancy status (multigravida and primigravid) and the vaginal microbiome, PERMANOVA, a distance-based analysis of variance method was used (999 within-subject permutations, “adonis” function in the R “vegan” package 1.17–4^54^. An Omnibus test which takes the minimum of the PERMANOVA p-values of individual β-diversity measures as test statistic, was used to combine association evidence from different β-diversity measures and an overall association p-value was reported (“PermanovaG” function in the R “GUniFrac” package v1.1)^55^. Ordination plots were generated using classic multi-dimensional scaling (MDS) in R with the command described (“cmdscale” function in the R “stats” package). Significance at differences in taxa abundance between pregnancy and postpartum samples was assessed using normalized abundance data at each taxonomic rank (phylum, class, order, family, genus and ASV level) using a permutation test (999 within-subject permutations)^55^. The count data was normalized by dividing by the Geometric Mean of Paired Rations (GMPR) size factor, which has been shown to be a robust estimation of the sampling effort^56^. The permutation test uses the F-statistic of a linear model (square-root normalized taxa abundance as the response variable) as the test statistic. Taxa with prevalence less than 10% or with a maximum proportion (relative abundance) less than 0.2% were excluded from testing to reduce the numbers of tests. Next, false discovery rate (FDR) control (Benjamini–Hochberg procedure)^57^ was used to correct for multiple testing at each taxonomic level and FDR- adjusted p-values or q-values < 0.10 were considered significant. Similar permutation tests were used to identify stable taxa, whose within-subject variability was smaller than the between-subject variability by treating the subject ID as a covariate in the permutation test.

### Ethics approval and consent to participate

The research was performed in accordance with the principles of the Declaration of Helsinki. Ethical approval for this study was obtained in accordance with the Nigerian National Code for Health Research Ethics and was approved by the Ethical Review Committee of the Joint University of Ibadan/University College Hospital Health Research Ethics Committee (UI/UCH HREC) with Registration Number NHREC/05/01/2008a and IRB approval number UI/EC/18/0411. All experiments were performed in accordance with the approved guidelines. Written informed consents, which included “Informed consent form for scientific research and information collection,” have been signed by all participants.

## Results

### Participants population

The protocol for this study was approved by the University of Ibadan/University College Hospital Joint Ethics Committee (Registration Number NHREC/05/01/2008a) with IRB number UI/EC/18/0411. A total of 38 women were recruited into the study. Of these, 16 attended for sampling at the second time point, 15 attended at the third timepoint and 19 women attended for post-natal sampling. Further sociodemographic and clinical data of participants are summarized in Table S1. Data presentation of participants across all sampling timepoint during pregnancy and postpartum is illustrated in the flow chart in Figure S2.

### Characterization of vaginal bacterial communities

**Figure 1:**
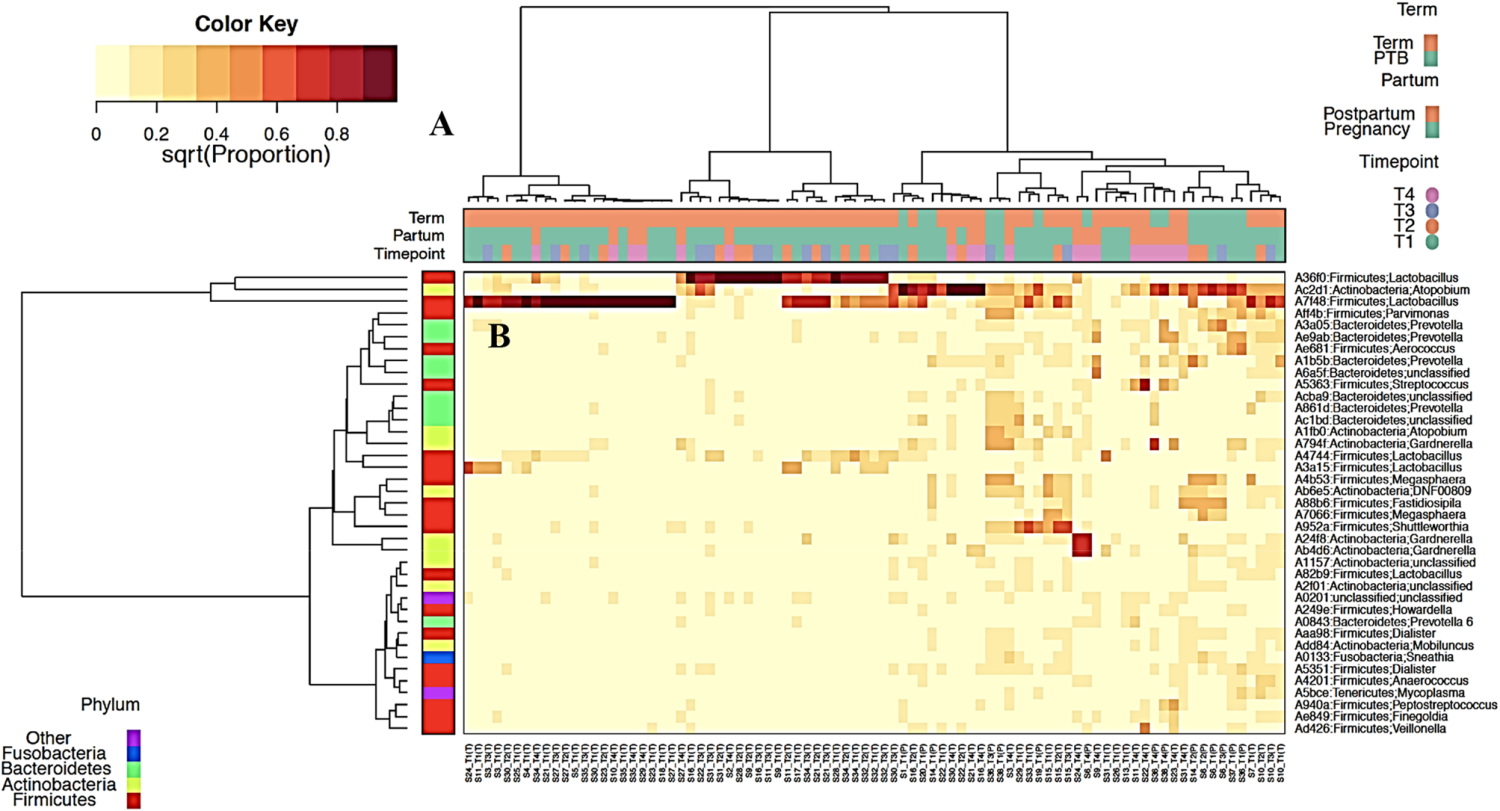
Bacterial species composition of vaginal community state types (CST) during pregnancy and postpartum. **(A)** Hierarchical clustering based on amplicon sequence variant (ASV) abundance, Hellinger distance (Euclidean distance on square-root proportion data), Wald. D linkage reveals microbiome from a Nigerian cohort can be clustered into 4 community clusters. **(B)** Heatmap of relative abundance of amplicon sequence variants characterizing the community state types represented. Figure created with R using the R ‘GMD’ package version 0.33. https://www.rdocumentation.org/packages/GMD/versions/0.3.3.

Hierarchical clustering analysis of bacterial species from pregnancy and postpartum vaginal microbial communities in Nigerian women revealed four major community state types (Fig. 1): CST I (*L. crispatus* dominated), CST II (*L. gasseri* dominated), CST III (*L. iners* dominated), and CST IV (non-*Lactobacillus* dominated). Pregnant participants show a higher frequency of CST III, followed by CST IV, CST I then CST II while majority of postnatal women have higher frequency of CST IV followed by CST III and CST I. *L. johnsonii* was found dominant only in one woman during pregnancy. Among pregnancy vaginal samples dominated with CST IV (27.9%), few participants (10.3%) that delivered at term had CST IV vagitypes while the PTB group (17.6%) had vaginal samples enriched with CST IV vagitypes. Overall, CST IV vagitypes were significantly overrepresented in postnatal women compared to pregnant women (p_*Fisher exact test*_ = 0.0380). Frequencies of observed CST across pregnancy and postpartum vaginal samples are described in detail (Table 1).

**Table 1:** Frequencies of observed community state type across pregnancy and postpartum vaginal samples and generalized mixed effect model with subject-level random intercept exploring the association between CSTs and status (pregnancy and postpartum).

Vagitypes	Overall (frequency)	Pregnancy	Postpartum	p-values
CST I (*L. crispatus*)	17/87 (19.5%)	16/68 (23.5%)	1/19 (5.3%)	10.0E−01
CST II (*L. gasseri*)	3/87 (3.4%)	3/68 (4.4%)	0/19 (0%)	7.0E−02
CST III (*L. iners*)	31/87 (35.6%)	27/68 (39.7%)	4/19 (21.1%)	**2.0E−02**
CST IV	33/87 (40.2%)	19/68 (27.9%)	14/19 (73.7%)	**5.6E−06**

The results indicate that the prevalence of CST-IV is significantly increased postpartum.

Significant p-values are depicted in bold face.

### Microbiome transitions and stability during pregnancy

**Figure 2:**
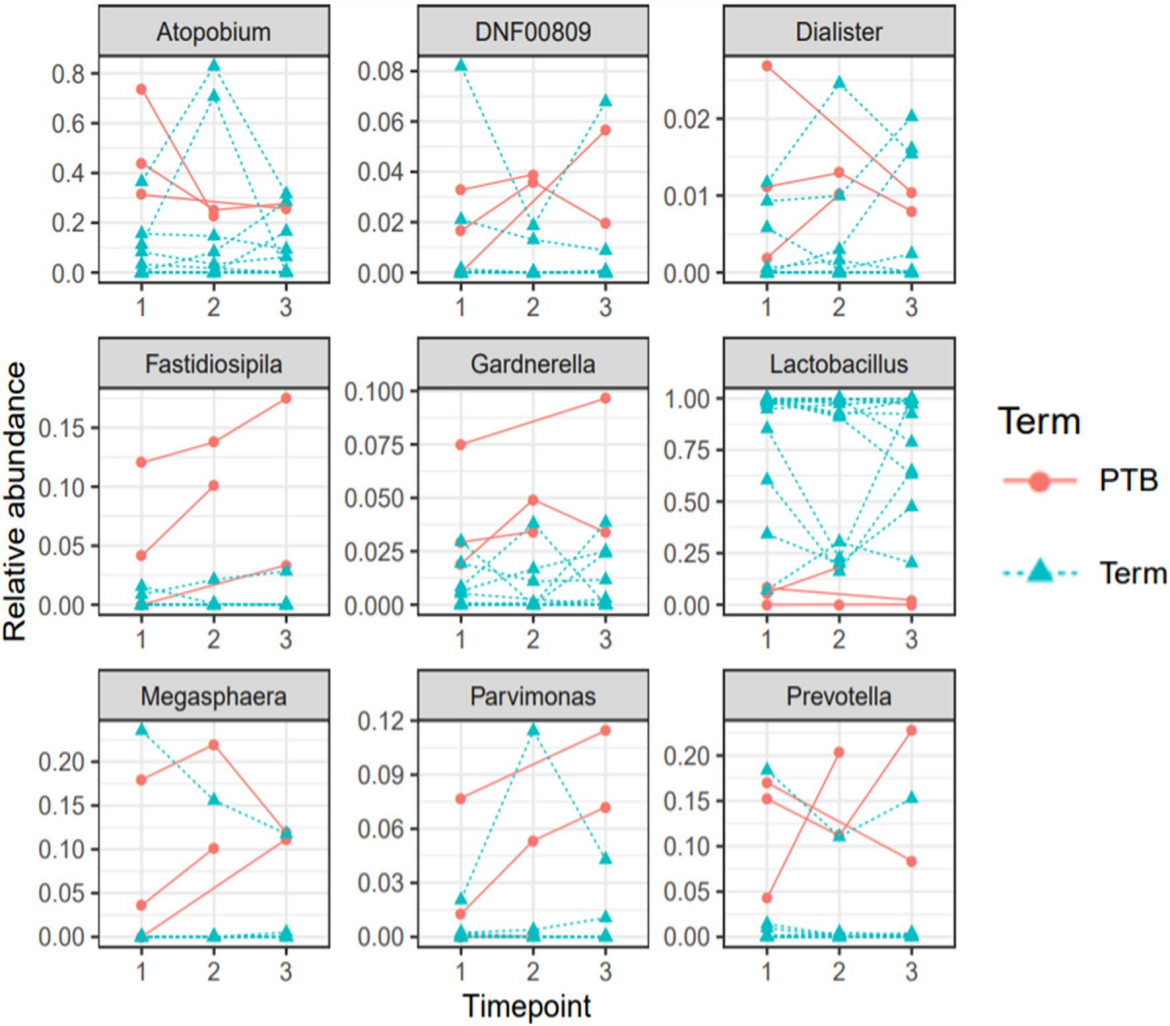
Longitudinal relative abundance of various taxa during preterm and term pregnancies.** (A)** The vaginal microbiome within a subject were significantly less likely to switch to another vagitype than between subjects (q < 0.01, Permutation test)**.** Each timepoint represent gestational weeks of pregnancy; timepoints 1 (17–21 gestational weeks), timepoint 2 (17–21 gestational weeks) and Time point 3 (17–21 gestational weeks).

Stability is a measure of how bacterial communities persist in the same CST over time without switching to another CST. A high level of stability is depicted by the prevalence of a vagitype over time whereas low level of stability is depicted by vagitypes that switch to a different community state type overtime. Vaginal microbiomes were relatively stable within-subject compared to between-subjects (q < 0.01, Permutation test) (Fig. 2). Between first and second timepoint during pregnancy, the vaginal CST I (*L. crispatus* dominated) remained stable but transitioned to CST IV-B in one participant. A similar trend was equally observed with CST II (*L. gasseri* dominated) and CST III (*L. iners* dominated) (Table S2). Tracking the non-lactobacillus dominated vagitypes (CST IV), our result show that CST IV vagitypes observed at the first sampling timepoint remained persistent at subsequent visits. In particular, vaginal samples of participants who delivered preterm were dominated by *Atopobium vaginae* from the first sampling time point and remained persistent until preterm delivery.

Further, to demonstrate how stable individual microbial taxa were across the pregnancy sampling time points, we performed the Augmented Dickey Fuller (ADF) test, which evaluated the null hypothesis that a given ASV is non-stationary (that is, the ASV tends to return to an equilibrium value). *L. crispatus*, A. *vaginae,* and *L. iners* were stationary according to the ADF test (P < 0.05). In particular, *L. iners* had the highest index of stability from our ADF model (Table 2).

**Table 2:** Index of stability from augmented Dickey Fullers test.

Vagitype	Stationary	P value
*Atopobium vaginae*	Yes	**5.35E−06**
*Lactobacillus crispatus*	Yes	**2.70E−12**
*Lactobacillus iners*	Yes	**1.54E−15**

Significant p-values are depicted in bold face.

### Trend in diversity across longitudinal vaginal samples in term and preterm group

**Figure 3:**
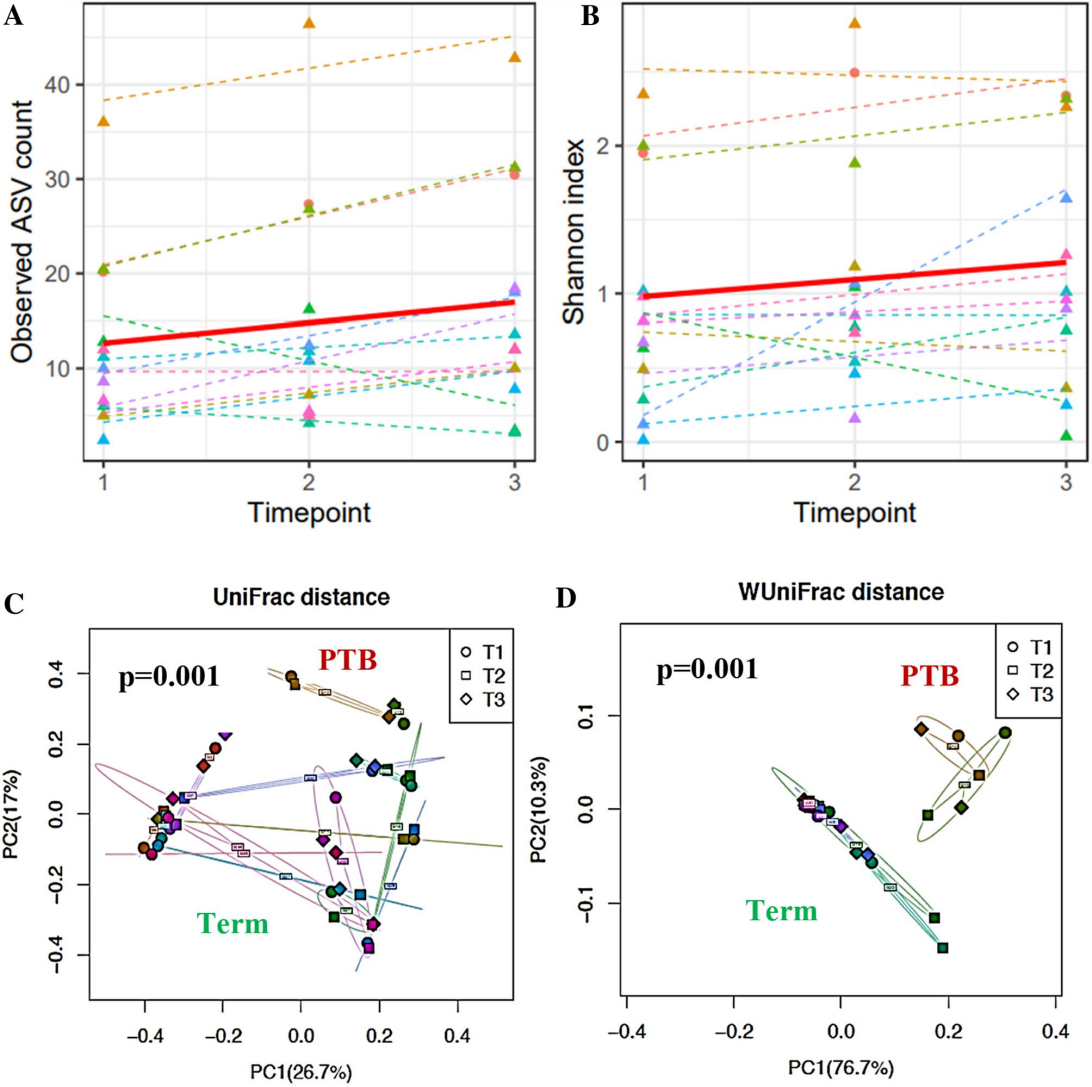
Longitudinal trends in diversity of the vaginal microbiome with increasing gestational age. Each timepoint represent gestational weeks of pregnancy; timepoints 1 (17–21 gestational weeks), timepoint 2 (27–31 gestational weeks) and Time point 3 (17–21 gestational weeks). **(A,B)** Alpha diversity increased with increasing gestational age (p = 0.006 and p = 0.07; LMM) for observed amplicon sequence variant (ASV) and Shannon diversity respectively. Each color represents a participant. Preterm birth (PTB participants are the top two participants depicted with light lemon and orange dash line. The dash line indicates the individual trend while the solid line indicates the overall trend (population-level trend). **(C)** Longitudinal beta diversity show separate community structure across term and preterm group **(**unweighted UniFrac). **(D)** Longitudinal beta diversity show separate community structure across term and preterm group **(**weighted UniFrac). Time points are identified with shapes (Circular, Timepoint 1; Square, Timepoint 2; Sphere, Timepoint 3).

In our Nigerian cohort, we observed a significant trend of increasing α-diversity with increasing gestational age. At the first time point (middle trimester), a trend of lower α-diversity of the vaginal microbiome was observed in the majority of women who delivered at term whereas a higher α-diversity was observed at second and third timepoints (p = 0.006 and 0.07; LMM for Observed ASV and Shannon diversity respectively) (Fig. 3A,B).

The microbiome structure of the preterm group across pregnancy follows a predictive pattern of dissimilarity in community composition which mirrors the dichotomy observed in the community structure. Figure 3 illustrates a longitudinal β-diversity measure across pregnancy samples (term and preterm samples) which shows different community structures across term and preterm groups (p = 0.001, PERMANOVA) for both weighted and unweighted UniFrac measure. Principal coordinate analysis also shows clear separation between the microbial communities of term and preterm delivery groups across samples collected longitudinally (Fig. 3C,D).

### Postpartum alters vaginal microbiome profiles

**Figure 4:**
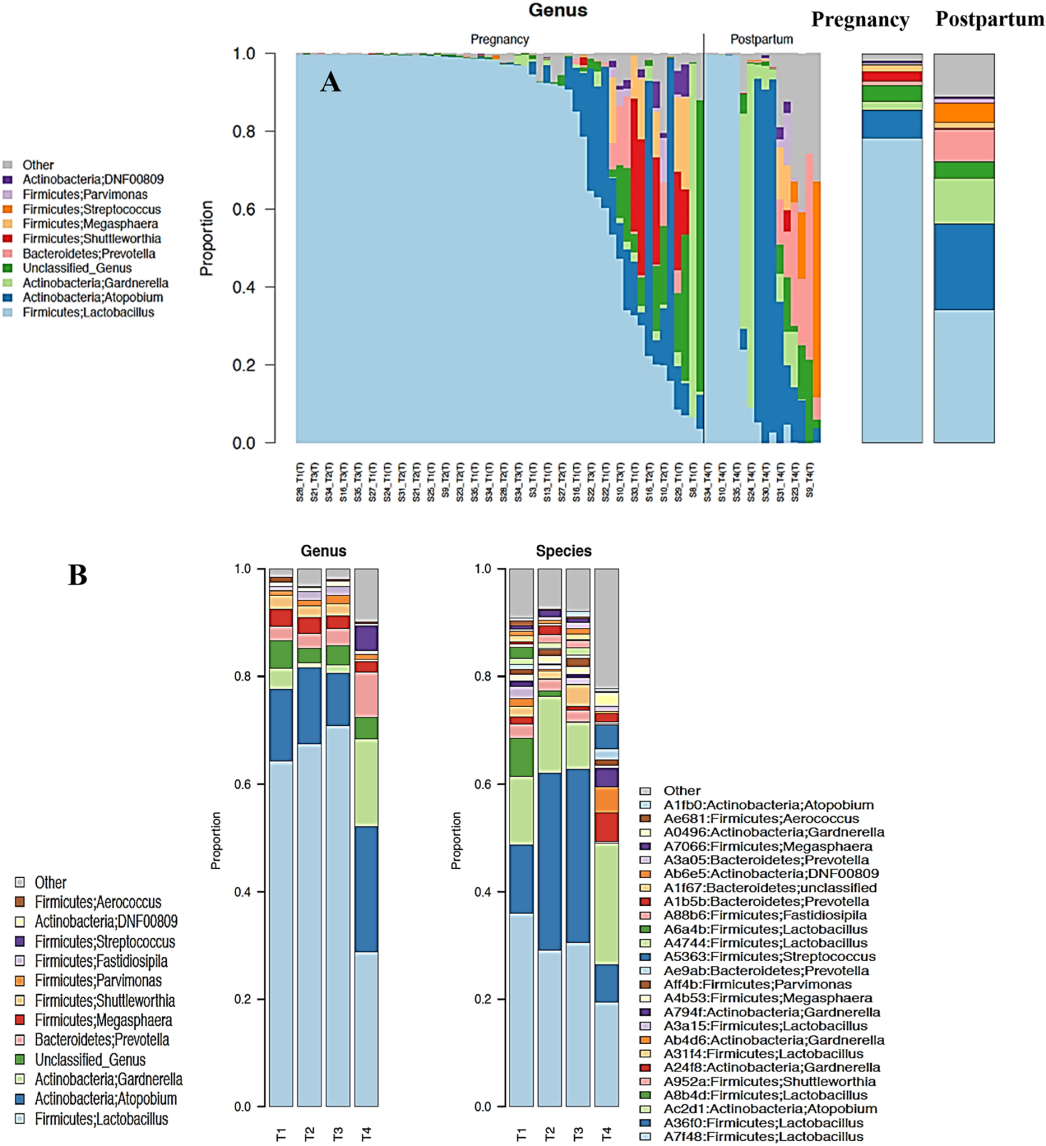

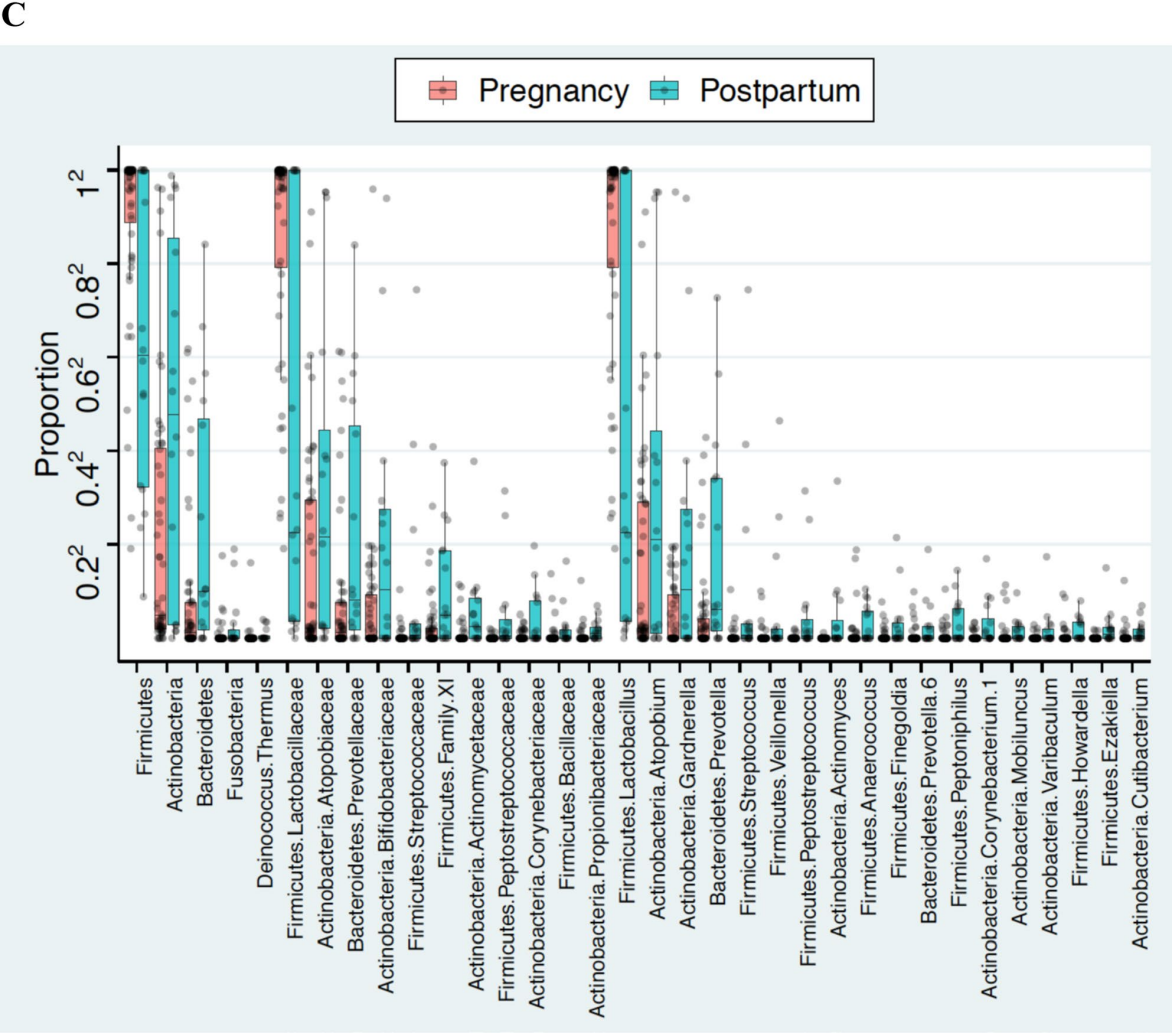
Vaginal Profile and taxa abundance analysis across pregnancy and postpartum vaginal samples. **(A)** Stacked bar plots of average relative abundance at genus taxonomic level compared across pregnancy and postpartum vaginal samples. Vaginal profile of pregnant women were *Lactobacillus* dominated compared to postpartum vaginal samples with a high proportion of abnormal vagitypes including unidentified bacteria, *Prevetolla*, *Streptococcus*, *Gardnerella*, and *Atopobium*. **(B)** Stacked bar plots of average relative abundance at genus taxonomic level compared according to gestational age of delivery and postpartum. With increasing gestational age, pregnant women were also more likely to steadily maintain a Lactobacilli profile, this however changed at the postpartum to become less dominated by *Lactobacillus* and more dominated by community state type IV both at genus and specie level. **(C)** Tukey boxplots show differences in relative abundance at phylum, family and genus level across pregnancy and postpartum samples. Firmicutes, Lactobaccillaceae, and *Lactobacillus* dominate the pregnancy vaginal samples compared to the postpartum samples dominated by Bacterial vaginosis associated bacteria.

The vaginal profile during pregnancy was dominated by *Lactobacillus* and commensurately lower level of other non-*Lactobacilli* microbiome profile while postpartum samples were characterized by minute proportions of *Lactobacillus* species and an increased dominance of bacterial vaginosis associated bacteria (BVAB) (Fig. 4A). With increasing gestational age, pregnant women were also more likely to steadily maintain a *Lactobacilli* profile (Fig. 4B).

A taxa analysis of these profiles confirmed a significant shift in the postpartum microbiome. While pregnancy was associated with a microbiome largely dominated by Firmicutes (*Lactobacillus*), puerperium was characterized by a shift in bacteria phylum structure to become dominated by anaerobes especially Actinobacteria (*Gardnerella* and *Atopobium*) (Fig. 4C).

### Increased vaginal microbiota richness and diversity at postpartum

**Figure 5:**
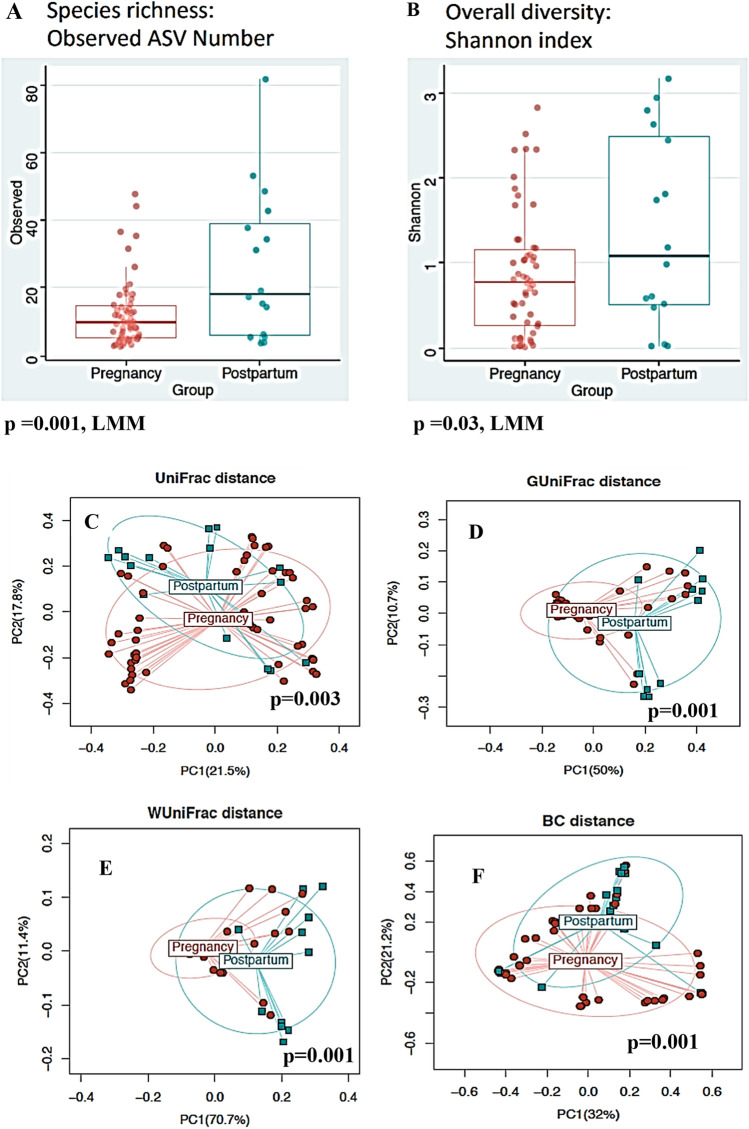
α- and β-diversity of vaginal microbiome of participants’ pregnancy and postpartum vaginal samples. **(A)** α-diversity computed with observed amplicon sequence variants **(B)** Shannon diversity index metrics. Participants group are identified with colored dots within bar boxes (light red-postpartum; pine green-term). Postpartum group shows higher diversity and greater number of amplicon sequence variants (ASV) compared to term group. β-diversity, based on **(C)** unweighted, **(D)** generalized, **(E)** weighted and **(F)** Bray Curtis UniFrac distance. Participants group are identified with color and shape within a circle (circular red, preterm birth squared pine green, term). A significant separation between the term and preterm birth microbiome community was observed (p unweighted = 0.003, p generalized = 0.001, p weighted = 0.001, p Bray Curtis = 0.001 respectively; Omnibus test).

According to α-diversity, the observed amplicon sequence variants (ASVs) and Shannon index were significantly higher in the postpartum vaginal samples compared to pregnancy samples (p = 0.001 and 0.05 respectively, LMM) (Fig. 5A,B). With respect to β diversity, principal coordinate analysis also shows clear separation between the microbial communities of pregnancy and postpartum samples (Fig. 5C). This separation is consistent across all the β diversity measures (Fig. 5D–F).

### Shift in vaginal microbiomes at postpartum

**Figure 6:**
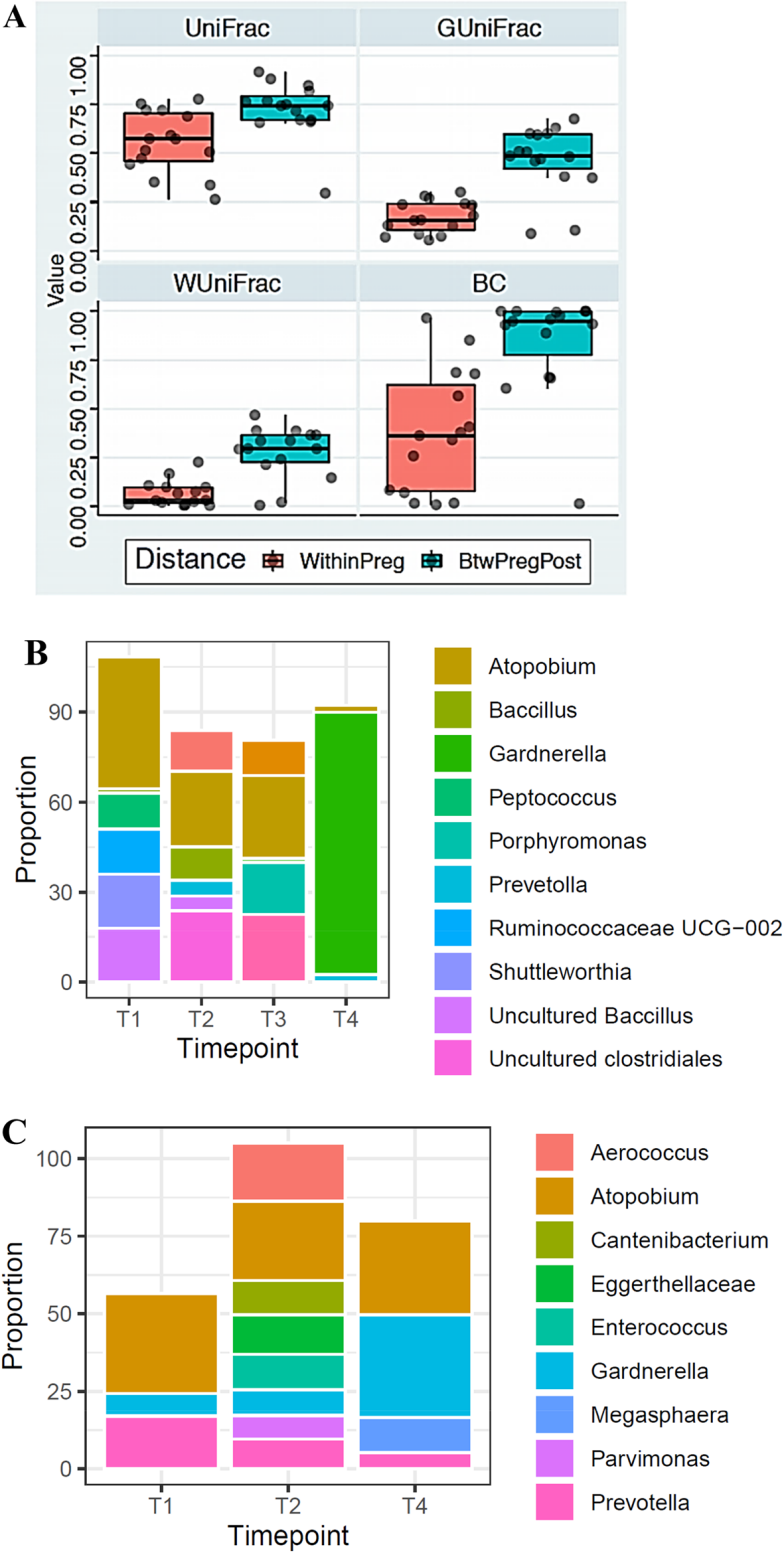
Shift in vaginal microbiome within pregnancy and between pregnancy (term and preterm birth) and postpartum vaginal samples. **(A)** A compositional dramatic shift in the postpartum vaginal microbiomes was observed such that the distance between samples during pregnancy is much smaller than the distance between pregnancy and postpartum. Wilcox paired test p = 0.008, 0.0002, 6e−5, 0.003 for UniFrac, GUniFrac, WUniFrac and BC distance, respectively. **(B)** *Atopobium vaginae* persistent in participant with late preterm birth throughout gestation but changed during the postpartum to become dominated by *Gardneralla vaginalis*. **(C)** *Atopobium vaginae* persistent in participant with early preterm birth throughout gestation but switched at postpartum to become dominated by *Gardneralla vaginalis*.

All CST I switched to CST IV, while other CSTs showed more resilience to change (Table S3). We also show herein that changes between pregnancy timepoints were much smaller than changes observed at postpartum. This difference reached statistical significance for all the measures described (Fig. 6A). Notably, this shift in microbiome at postpartum was equally observed with women who delivered preterm. The vaginal microflora of four PTB participants dominated by *Atopobium* during pregnancy persisted with increasing gestational age. However, we observed a compositional shift from *Atopobium vaginae* (CST IV) to *Gardnerella vaginalis* (CST IV) in PTB subjects who returned for postpartum sampling (Fig. 6B,C).

### Association between vaginal microbiota and maternal covariates

With our sources of variability model, we explored the relationship between the vaginal microbiota and steroid hormones, and identified factors that could impact the vaginal microbiome while adjusting for potential confounders. Age (women below 35 years old or women above 35 years old), pregnancy status (multigravid or primigravid) and delivery mode (caesarian section or vaginal delivery) were not significantly associated with the vaginal microbiota whereas maternal estradiol level (p_estradiol_ = 0.006, Omnibus), and maternal progesterone level (p_progesterone_ = 0.001, Omnibus) were significantly associated with the vaginal microbiome (Table 3).

**Table 3:** Results from PERMANOVA-based omnibus test combining different distance metrics comparing the association between the vaginal microbiota and clinical variables.

Clinical variables	UniFrac	GUniFrac	WUniFrac	BC	Omnibus P-value
Progesterone level	0.426	0.162	0.171	0.001	**0.001**
Estradiol level	0.654	0.056	0.055	0.003	**0.006**
Pregnancy status (multigravid vs primigravid)	0.442	0.079	0.079	0.289	0.186
Age (women above 35 years vs women below 35 years)	0.137	0.332	0.319	0.914	0.302
Delivery mode (ceaserian section vs vaginal delivery)	0.435	0.26	0.253	0.180	0.377

Significant p-values are depicted in bold face.

### Dynamics of the vaginal microbiome with steroid hormones

**Figure 7:**
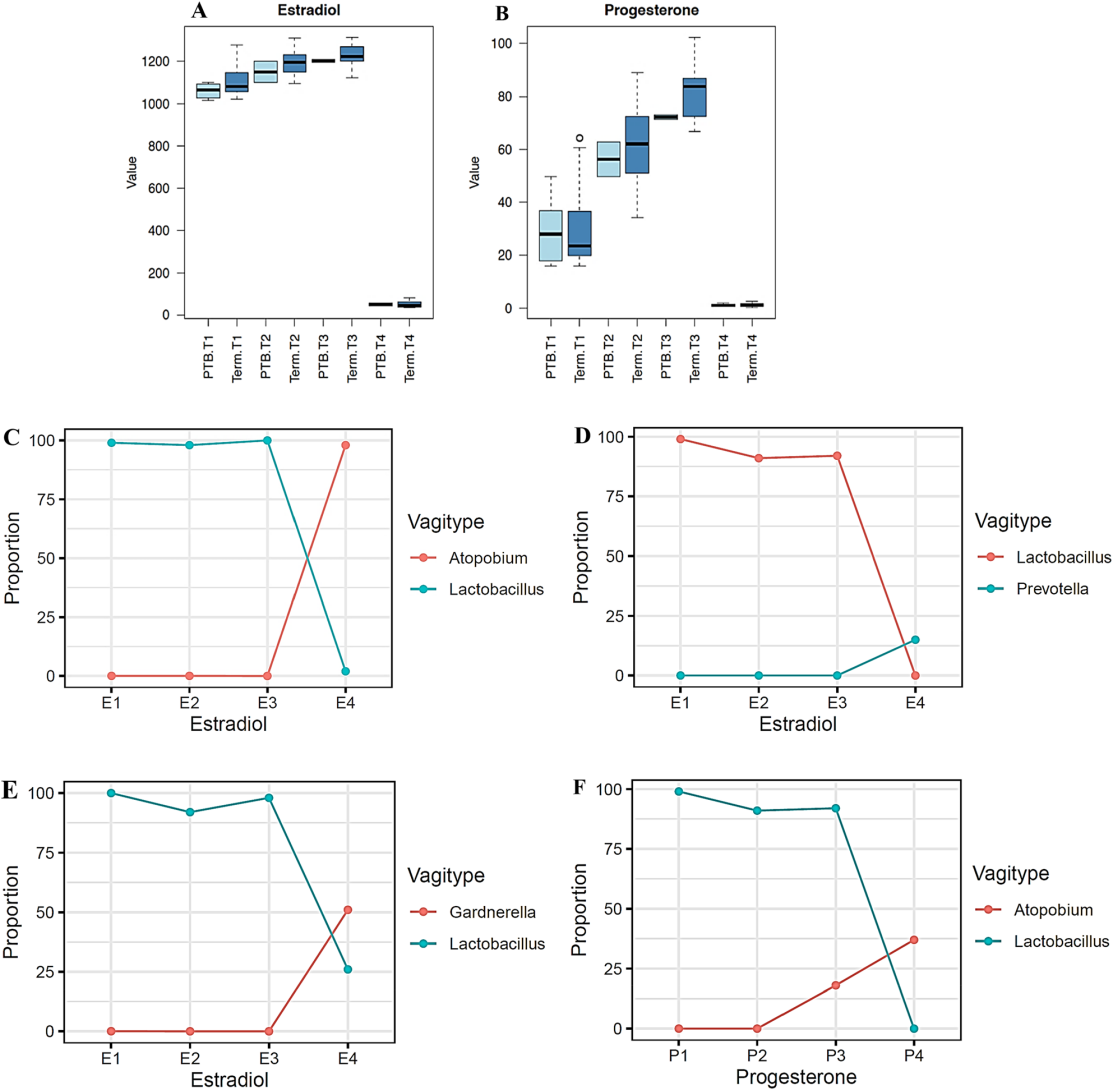

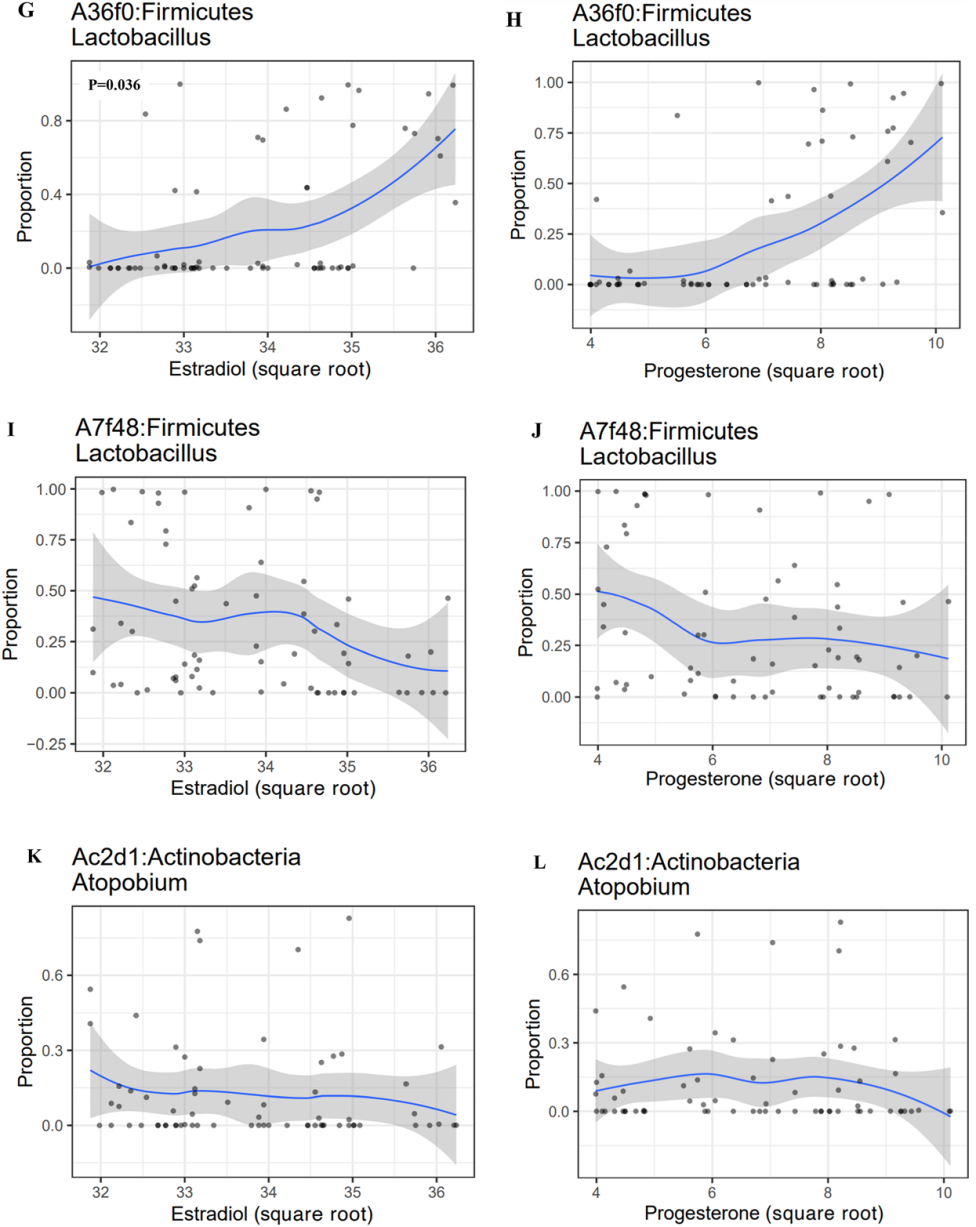
Dynamics of the vaginal microbiome with hormones in pregnant and postnatal women. Hormone concentration significantly increased throughout pregnancy and reduced at the postpartum (p = 0.0001, LMM). **(A)** Estradiol concentration across all participants during pregnancy (term and preterm) and postpartum. **(B)** Progesterone concentration across all participants during pregnancy (term and preterm) and postpartum. Across all sampling points, participant that delivered preterm were associated with lower level of estradiol with a value tending towards significance (p = 0.077, LMM), while the preterm birth group were not associated with low progesterone level (p = 0.66, LMM). The vaginal microbiome dominated by *Lactobacillus* as hormone increases but switches to bacterial vaginosis associated bacteria vagitype with a drastic fall in hormone concentration at the postpartum. **(C)** Pregnancy *Lactobacillus* dominated vagitype switched to *Atopobium* postpartum with a drastic fall in estradiol concentration (E1, E2, E3 connotes estradiol concentration at 17–21 weeks, 27–31 weeks, 37–41 weeks respectively, E4 connotes estradiol concentration at postpartum) **(D)** Pregnancy *Lactobacillus* dominated vagitype switched to *Prevotella* with a decrease in estradiol concentration postpartum (E1, E2, E3 connotes estradiol concentration at 17–21 weeks, 27–31 weeks, 37–41 weeks respectively, E4 connotes estradiol concentration at postpartum). **(E)** Pregnancy *Lactobacillus* dominated vagitype switched to *Gardnerella* with a drastic fall in estradiol concentration postpartum(E1, E2, E3 connotes estradiol concentration at 17–21 weeks, 27–31 weeks, 37–41 weeks respectively, E4 connotes estradiol concentration at postpartum) **(F)** Pregnancy *Lactobacillus* dominated vagitype switched to *Atopobium* with a decrease in progesterone concentration(P1, P2, P3 connotes estradiol concentration at 17–21 weeks, 27–31 weeks, 37–41 weeks respectively, P4 connotes estradiol concentration at postpartum). **(G)** Longitudinal association between *L. crispatus* and steroid hormones show that with increasing gestational age and increasing hormone concentration, the abundance or proportion of *L. crispatus* (designated with amplicon sequence variant number *A36f0)* concurrently increased throughout pregnancy with increasing estradiol level (p value = 0.036, permutation test). **(H)** *L. crispatus* proportions increased concurrently with increasing progesterone level (p value = 0.034, permutation test). **(I,J)** *L. iners* (designated with ASV number *A7f48)* and **(K,L)***, A. vaginae* (designated with ASV number *Ac2d1*) were not significantly associated with increasing estradiol and progesterone concentration throughout pregnancy (p value > 0.05, permutation test) as the proportions of *L. iners* and *A.vaginae* reduced with increasing hormone concentration.

Hormone concentrations significantly increased throughout pregnancy and reduced postpartum (p < 0.001, LMM) (Fig. 7A,B). Commensurately, with a shift in hormonal milieu (both estradiol and progesterone concentration) pregnancy samples dominated by high proportions of *Lactobacillus* depleted to become dominated by BVAB (p_Lactobacillus_ < 0.001 and p_CST IV_ < 0.001; *spearman correlation test*) for both estradiol and progesterone respectively (Fig. 7C–F, Figure S3A–D). As estradiol and progesterone concentration increase during pregnancy, we observed an increasing trend in non-Lactobacillus dominated microbes (Figure S4A–D). This trend, however, did not apply to all participants with *Lactobacillus* dominated vagitypes. Conducting a longitudinal (within subject) association of the vaginal microbiome and hormones with our permutation model, we observed that the proportion of *L. crispatus* increased commensurately with increasing hormone concentration (p_estradiol_ = 0.036, p_progesterone_ = 0.034; permutation test) (Fig. 7G,H) while other vagitypes including *L. iners, and A. vaginae* did not (Fig. 7I–L).

### Relationship between postpartum vaginal microbiome and delivery mode

**Figure 8:**
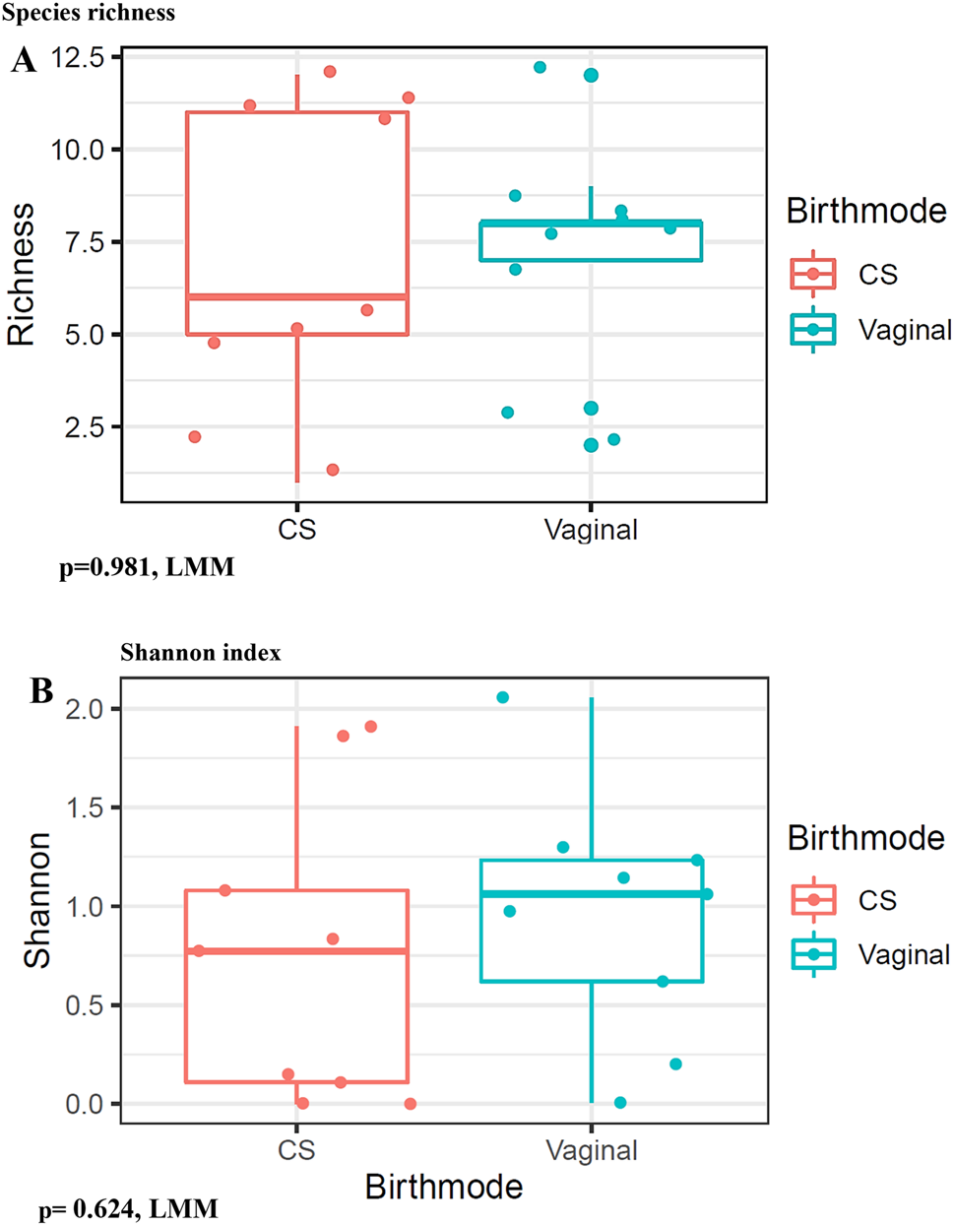
Relationship between postpartum microbiota and delivery mode. **(A)** Box plot show no significant evidence for impact of delivery mode on microbiome (p _richness_= 0.981, LMM), (p_shannon_ = 0.624, LMM).

To determine the association between postpartum vaginal microbiome and delivery mode, we explored the postpartum microbiome of women that delivered via Ceasarian section and vaginally. We demonstrate that delivery mode had no significant impact on the vaginal microbiome. (p_richness_ = 0.981, LMM) (p_shannon_ = 0.624, LMM) (Fig. 8A,B).

## Discussion

Here we present an exploratory, high-throughput and longitudinal assessment of the vaginal microbiome during pregnancy and postpartum in a Nigerian cohort. The dominant taxon in the vaginal microbiome of pregnant Nigerian women that delivered at term is *Lactobacillus.* The findings support the hypothesis that having a *Lactobacillus* spp.-dominated vagitype is strongly associated with healthy pregnancy outcomes^1,2,6,22^. As shown here and in previous studies^1,3,29^ in pregnancy, a larger proportion of women with healthy pregnancy outcome have *Lactobacillus* spp. in high relative abundance in the vaginal microbiota, whereas the majority of women lacking *Lactobacillus* spp. in the vaginal microbiota delivered preterm as also reported in previous studies^7,22,58,59^. In particular, *Atopobium* (CST IV) remained persistent throughout pregnancy in women who delivered preterm, buttressing a cause-effect relationship between *Atopobium* and preterm birth^7,60–62^. Our data further reveal that the vaginal microbiome of Nigerian women was typically dominated by *L. iners*. This lends important clues to the question of whether *L. iners* is indicative of a healthy or dysbiotic vaginal microbiota^63,64^. The data presented here is consistent with studies which support the notion that *L. iners* is part of a healthy microbiota, as most women in our cohort with *L. iners*-dominated vagitype delivered at term.

Consistent with previous studies^2,65^, the structure and composition of the vaginal microbiome at postpartum are significantly altered to comprise mostly of CST IV vagitypes. This variation could be explained by the presence of lochia (alkaline vaginal discharge during postpartum period)^66^. A major mechanism by which *Lactobacillus* restricts the growth of possible pathobionts is by creating an acidic vaginal microenvironment^12,13^. This led to the hypothesis^2^ that lochia alkaline discharge tends to impede *Lactobacillus* spp. growth and increase the propensity for several CST IV microbes to thrive.

As shown in other studies^2,29,65^, our data reveals a shift in the core structure and composition of the pregnancy vaginal microbiome during the postpartum period. This shift has led to the hypothesis^26,27^ that hormones drive the vaginal microbiome in pregnancy. While a compelling notion, delivery comes with many confounding factors. Our work verified this trend using time-longitudinal statistical associations during pregnancy, thereby assessing the role of hormones without the confounders of delivery. Our results support the notion that estrogen and progesterone enhance the presence of *L. crispatus,* but not other *Lactobacilli* species. This supports the notion that sex steroids steer the maturation of the vaginal epithelium giving rise to accumulation of glycogen^67^, which is then catabolized to products including maltose, maltriose, and maltatetraose by alpha amylase present in the host vaginal mucosa and that continuously supports *Lactobaccillus* colonization^68^. This finding further demonstrates that *L. crispatus* is a significant biomarker for healthy pregnancy progression^69^.

Our longitudinal analysis of the pregnancy vaginal microbiome revealed a higher initial microbiome diversity from some pregnant women in our cohorts compared to other reported populations^6,70^. Since this study is a pilot longitudinal assessment, we posit that the high initial microbiome diversity is individualized and requires further investigation with a larger cohort as we are limited by a small sample size. Overall, our analysis of the pregnancy and postpartum vaginal microbiome confirms that healthy pregnancy outcome is characterized by an enrichment of *Lactobacillus* spp. while PTB and puerperium vaginal microbiome is mostly characterized by CST IV microbes.

Large multicenter studies are needed to evaluate the scope of our findings. Undoubtedly there exists a relationship between age, parity and hormones as younger and primiparous women tend to have higher hormone concentration during pregnancy than older and multiparous women^71^. Further, some vagitype are more predominant in older women than younger women. For example, *Lactobacillus iners* was found dominate in women above 35 years compared to younger women^1^ however there was no significant association between both age, parity and the vaginal microbiome in this study. Indeed, a limitation of this study, is that direct matching and comparison of vagitypes across women of similar age, similar parity and similar hormone concentration were not obtained and thus it is difficult to establish a direct link between hormonal regulation of the vaginal microbiota across women of different age group and parity. This represents an important research question for future study. Nonetheless, our data merits consideration for studies investigating the pregnancy vaginal microbiome during pregnancy and postpartum and the relative contribution of and estradiol and progesterone concentration”.

In conclusion, we report a longitudinal study of the vaginal microbiome during pregnancy, puerperium and provide insights into the relative contribution of estradiol (E2) and progesterone (P1) in shaping of the vaginal microbiome during pregnancy and post-delivery in a Nigerian cohort. We provide evidence that the vaginal microbiome is associated with obstetric outcome. Our work shows that increasing hormone concentrations in pregnancy favors the dominance and high relative abundance of *L. crispatus* in the vaginal microbiota. Furthermore, the shift from a *Lactobacillus* dominated microbiome to a non-*Lactobacillus* dominated microbiome during postpartum is associated with a drastic fall in hormone levels. This has implications for future studies designed to explore relationships between the steroid hormones and vaginal microbiome in pregnancy, postpartum, and obstetric outcome.

## Supplementary Information

Supplementary Figure S1.

Supplementary Figure S2.

Supplementary Figure S3.

Supplementary Figure S4.

Supplementary Table S1.

Supplementary Table S2.

Supplementary Table S3.

## Data Availability

The metadata supporting this article has been provided and the sequence dataset supporting the results of this article has been publicly deposited and are available at the NCBI Sequence Read Archive, with BioProject accession no. PRJNA757760.

## Abbreviations

P1: Progesterone
E2: Estradiol
BV: Bacterial vaginosis
BVAB: Bacteria associated with vaginosis
ASV: Amplicon sequence variants
VMB: Vaginal microbiome
PTB: Preterm births
16S rRNA: 16S ribosomal RNA
CST: Community state types
EPTB: Early preterm birth
LPTB: Late preterm birth
ADF: Augmented Dickey Fuller test
