# Supplementary figures and images for: Lactobacillus crispatus thrives in pregnancy hormonal milieu in a Nigerian patient cohort
Source: Sci Rep. 2021 Sep 13;11:18152. doi: 10.1038/s41598-021-96339-y (PMC8437942; doi:10.1038/s41598-021-96339-y)

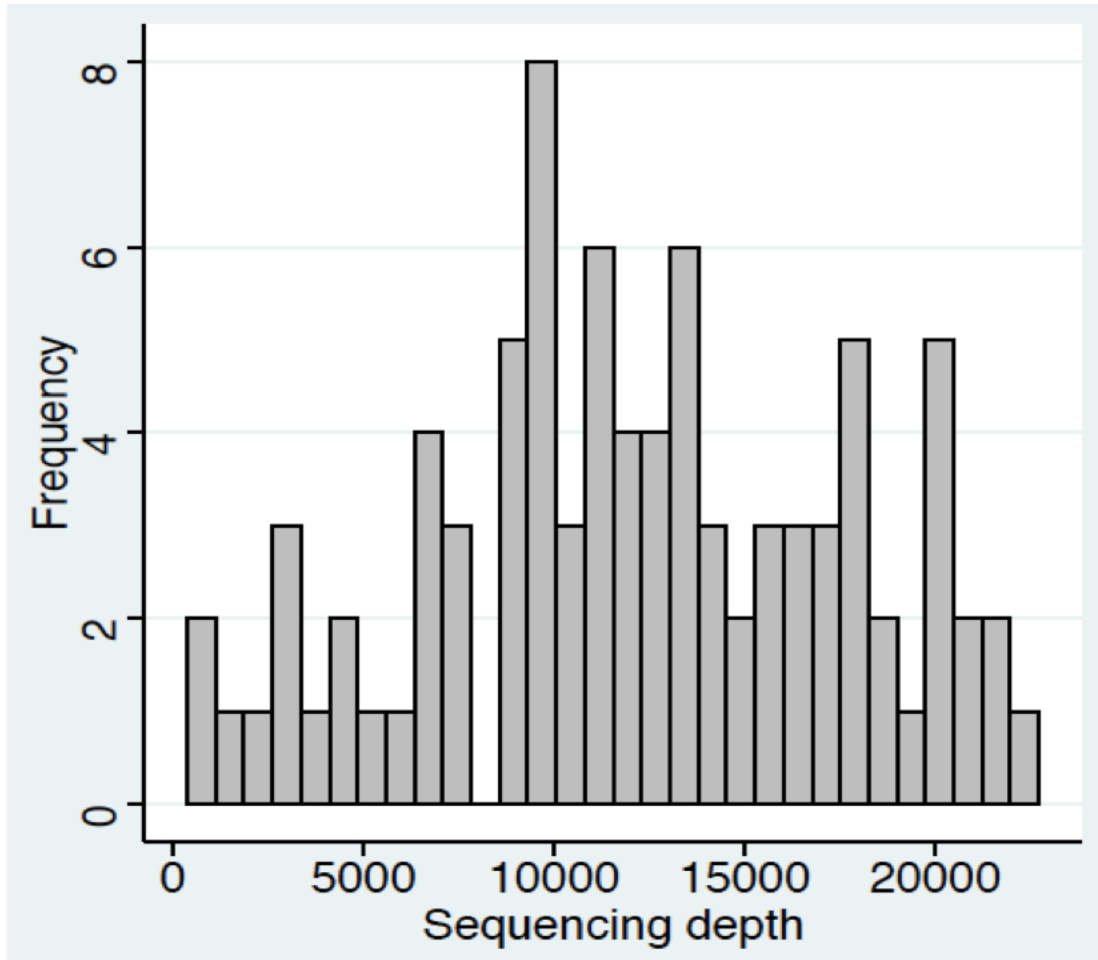

Figure S1. Sequencing Depth and Frequency of Sequences Across Rarefied Samples

Supplement: Supplementary file 1 — Supplementary Figure S1. [file 41598_2021_96339_MOESM1_ESM.pdf]
