# Supplementary material for: Lactobacillus crispatus thrives in pregnancy hormonal milieu in a Nigerian patient cohort
Source: Sci Rep. 2021 Sep 13;11:18152. doi: 10.1038/s41598-021-96339-y (PMC8437942; doi:10.1038/s41598-021-96339-y)

Figure S2: Prisma flow chart of data presentation of participants across all sampling timepoint during pregnancy and postpartum.

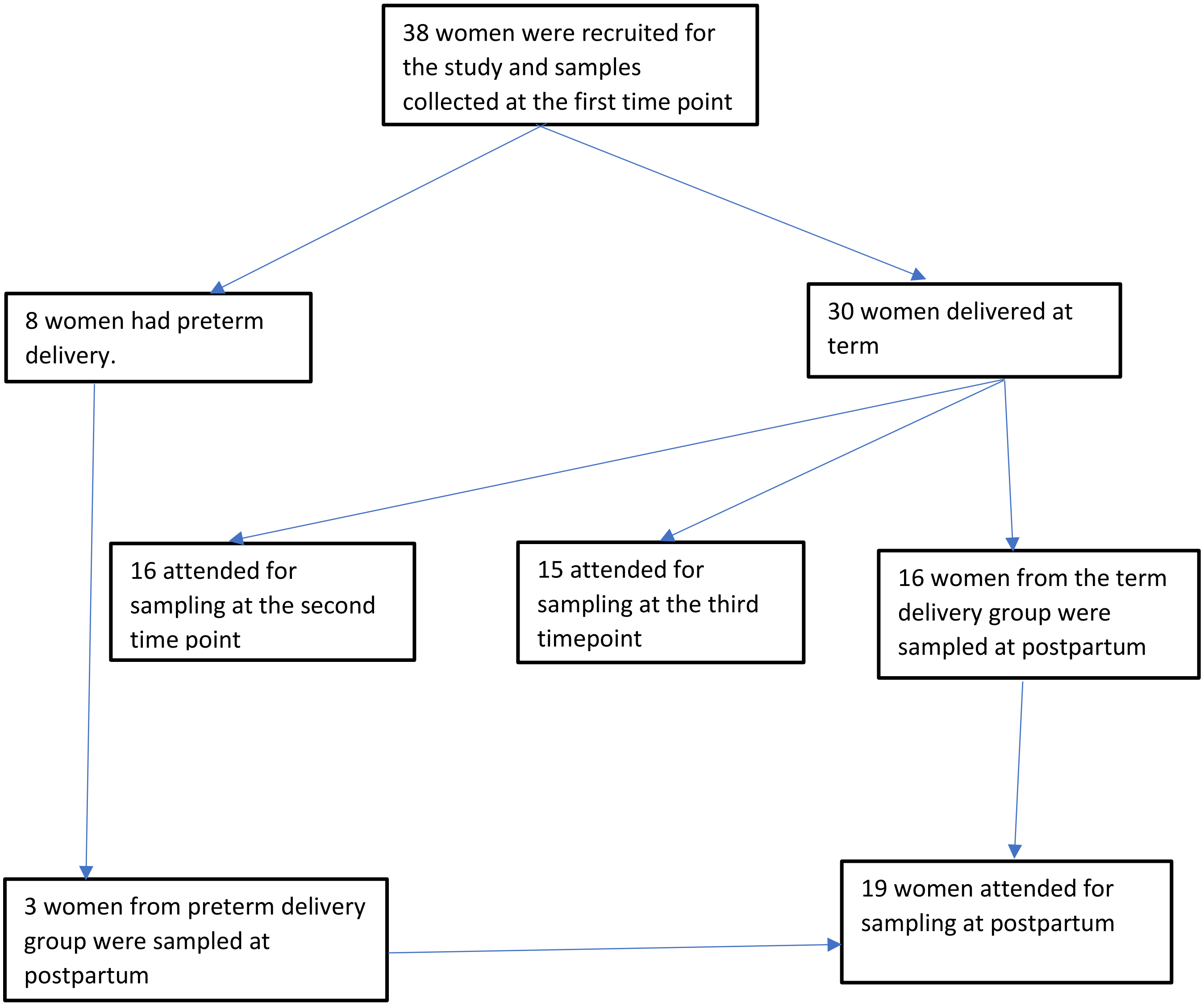

Supplement: Supplementary file 2 — Supplementary Figure S2. [file 41598_2021_96339_MOESM2_ESM.pdf]
