# Supplementary material for: Lactobacillus crispatus thrives in pregnancy hormonal milieu in a Nigerian patient cohort
Source: Sci Rep. 2021 Sep 13;11:18152. doi: 10.1038/s41598-021-96339-y (PMC8437942; doi:10.1038/s41598-021-96339-y)
Supplement: Supplementary file 3 — Supplementary Figure S3. [file 41598_2021_96339_MOESM3_ESM.pdf]

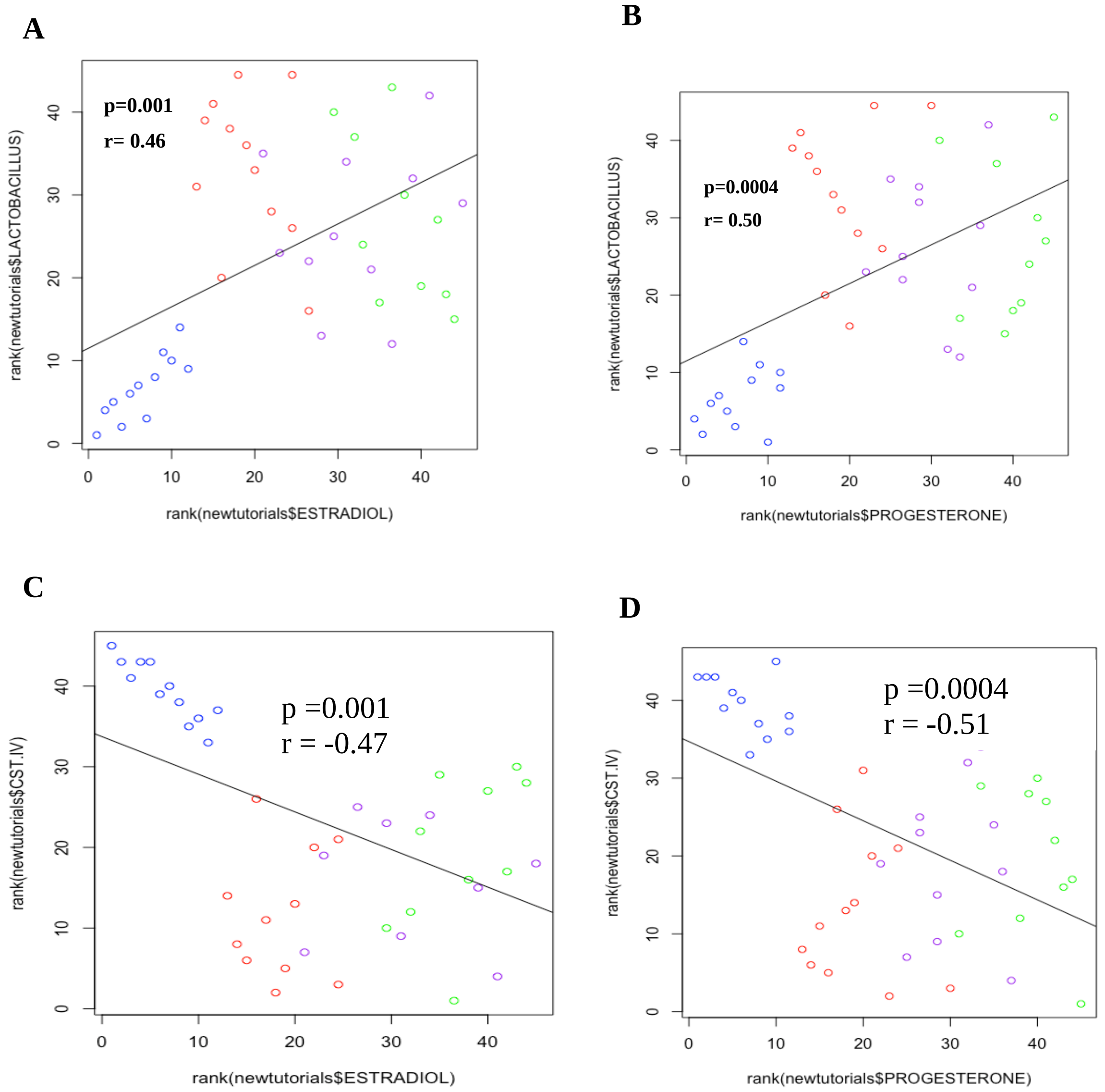

Figure S3 Dynamics of the vaginal microbiome with steroid hormones in pregnant and postnatal women. With a shift in hormonal milieu (both estradiol and progesterone concentration) pregnancy samples dominated by high proportions of *Lactobacillus* (A and B) depleted to become dominated by CST IV vagitypes ($p_{Lactobacillus}$ <0.001 and $p_{CST\ IV}$ <0.001; *spearman correlation test*).C and D, Low hormone concentration correlates with high proportions of CST IV and viceversa. Timepoints are identified with circular shape and color (Red, Timepoint 1; Purple, Timepoint 2; Green, Timepoint 3; and Blue, Timepoint 4).
