# Supplementary material for: Lactobacillus crispatus thrives in pregnancy hormonal milieu in a Nigerian patient cohort
Source: Sci Rep. 2021 Sep 13;11:18152. doi: 10.1038/s41598-021-96339-y (PMC8437942; doi:10.1038/s41598-021-96339-y)
Supplement: Supplementary file 4 — Supplementary Figure S4. [file 41598_2021_96339_MOESM4_ESM.pdf]

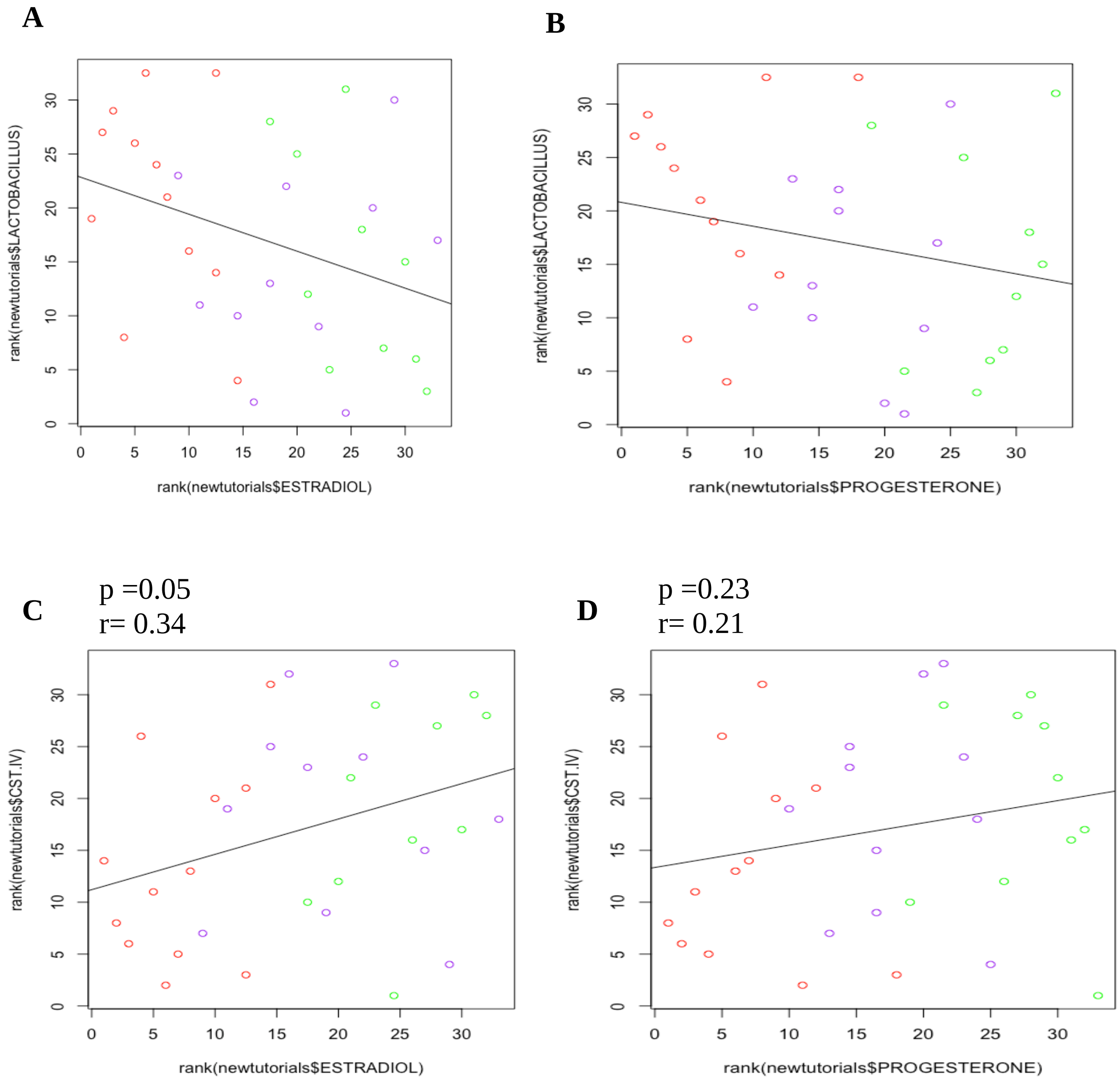

Figure S4 Dynamics of the vaginal microbiome with steroid hormones in pregnant women sampled longitudinally.

An increase in estradiol and progesterone concentration did not potentiate *Lactobacillus* drive as with increasing gestational age an increasing trend with BVAB microbes was observed instead of *Lactobacillus* **(A, B, C, D).** Timepoints are identified with circular shape and color (Red, Timepoint 1; Purple, Timepoint 2; Green, Timepoint 3).
