# Supplementary material for: Lactobacillus crispatus thrives in pregnancy hormonal milieu in a Nigerian patient cohort
Source: Sci Rep. 2021 Sep 13;11:18152. doi: 10.1038/s41598-021-96339-y (PMC8437942; doi:10.1038/s41598-021-96339-y)
Supplement: Supplementary file 6 — Supplementary Table S2. [file 41598_2021_96339_MOESM6_ESM.docx]

**Table S2 Community Transition and Stability Across Pregnancy Sampling Time Points**

|  | **First to Second timepoint (10 weeks interval)** | | | |  |
| --- | --- | --- | --- | --- | --- |
| **From** | **To** | **I (%)** | **II (%)** | **III (%)** | **IV (%)** |
| **I** |  | 4/18 (22.2) | 0 | 0 | 1/18(5.6) |
| **II** |  | 0/18(0) | 0 | 0 | 1 /18(5.6) |
| **III** |  | 1/18(5.6) | 0 | 7/18(38.9) | 0/18(0) |
| **IV** |  | 1/18(5.6) | 0 | 0/18(0) | 2/18(11.1) |
|  | **Second to Third timepoint (10 weeks interval)** | | | |  |
| **From** | **To** | **I (%)** | **II (%)** | **III (%)** | **IV (%)** |
| **I** |  | 3/12(0.3) | 0 | 1/12(8.3) | 0 |
| **II** |  | 0 | 0 | 0 | 0 |
| **III** |  | 0 | 0 | 5/12 (41.7) | 0 |
| **IV** |  | 1/12 | 0 | 0 | 1/12(8.3) |
|  | **First to Third time point (20 weeks interval)** | | | |  |
| **From** | **To** | **I (%)** | **II (%)** | **III (%)** | **IV (%)** |
| **I** |  | 0 | 0 | 0 | 0 |
| **II** |  | 0 | 0 | 0 | 0 |
| **III** |  | 0 | 0 | 2 /3(66.7) | 0 |
| **IV** |  | 0 | 0 | 0 | 1/3(33.3) |
