# Supplementary material for: Lactobacillus crispatus thrives in pregnancy hormonal milieu in a Nigerian patient cohort
Source: Sci Rep. 2021 Sep 13;11:18152. doi: 10.1038/s41598-021-96339-y (PMC8437942; doi:10.1038/s41598-021-96339-y)
Supplement: Supplementary file 7 — Supplementary Table S3. [file 41598_2021_96339_MOESM7_ESM.docx]

**Table S3 Community Transition across third pregnancy sampling timepoint and postpartum**

| **Last sampling during pregnancy to the puerperium by subjects** | | | | |  |
| --- | --- | --- | --- | --- | --- |
| **From** | **To** | **I (%)** | **II (%)** | **III (%)** | **IV (%)** |
| **I** |  | 0 | 0 | 0 | 6/19(31.6) |
| **II** |  | 0 | 0 | 0 | 0 |
| **III** |  | 0 | 0 | 3/19 (15.8) | 5/19 (26.3) |
| **IV** |  | 1/19(5.2) | 0 | 1/19(5.2) | 3/19 (15.8) |
|  |  |  |  |  |  |
